# Genomic Analysis of *Cutibacterium acnes* Strains Isolated from Prosthetic Joint Infections

**DOI:** 10.3390/microorganisms9071500

**Published:** 2021-07-14

**Authors:** Llanos Salar-Vidal, Yvonne Achermann, John-Jairo Aguilera-Correa, Anja Poehlein, Jaime Esteban, Holger Brüggemann, on behalf of the ESCMID Study Group for Implant-Associated Infections (ESGIAI)

**Affiliations:** 1 Clinical Microbiology Department, IIS-Fundación Jiménez Díaz, Av. Reyes Católicos, 2, 28040 Madrid, Spain; john.aguilera@fjd.es (J.-J.A.-C.); jesteban@fjd.es (J.E.); 2 Division of Infectious Diseases and Hospital Epidemiology, University Hospital of Zürich, Rämistrasse 100, 8091 Zürich, Switzerland; yvonne.achermann@usz.ch; 3 Faculty of Medicine, University of Zürich, Rämistrasse 100, 8091 Zürich, Switzerland; 4 Department of Genomic and Applied Microbiology, Institute of Microbiology and Genetics, University of Göttingen, 37077 Göttingen, Germany; apoehle3@gwdg.de; 5 Department of Biomedicine, Aarhus University, 8000 Aarhus, Denmark; brueggemann@biomed.au.dk

**Keywords:** *Cutibacterium acnes*, *Propionibacterium acnes*, prosthetic joint infection, phylotype, genome sequencing

## Abstract

*Cutibacterium acnes* is a common cause of prosthetic joint infections (PJIs). The *C. acnes* population can be divided into six main phylotypes (IA_1_, IA_2_, IB, IC, II and III) that are associated with different clinical conditions and normal skin. A single-locus sequence typing (SLST) scheme can distinguish ten main SLST types: A-E (all IA_1_), F (IA_2_), G (IC), H (IB), K (II), L (III). We genome-sequenced and compared 16 strains of *C. acnes* isolated from healthy skin (*n* = 4) and PJIs (*n* = 12), including six PJI cases with a good outcome (four shoulder PJIs, one hip PJI, one knee PJI) and six with infection relapse (three shoulder PJIs, three hip PJIs). The sequenced strains belonged to four different phylotypes (IA_1_, IA_2_, IB and II) and seven different SLST types. All five type IB strains (all SLST type H1) were PJI isolates (three hip PJIs, two shoulder PJIs), and four of these caused infection relapse (three hip PJIs, one shoulder PJI). Isolates from PJI cases with a good outcome belonged to three different phylotypes (IA, IB, II). Interestingly, four strains (three strains from PJI cases with good outcome and one strain from healthy skin) contained a linear plasmid; these strains belonged to different SLST types (A1, C1, F4, H1) and were isolated in three different hospitals. This study suggests that type IB strains have the potential to cause infection relapse, in particular regarding hip PJIs. Moreover, our study revealed that strains belonging to the same SLST type can differ in their accessory genome in different geographic locations, indicative of microevolution.

## 1. Introduction

Prosthetic joint infections (PJIs) are one of the most serious complications after joint replacement surgery. They are associated with high morbidity, mortality, and result in an increased economic burden to the healthcare system [1,2]. Treatment of PJIs often includes surgical intervention and prolonged antibiotic therapy depending on the causative agent [3]. Regardless of the advances in diagnosis and management [4], PJIs are challenging to eradicate. Treatment failure rates range from 0% to 40% [5,6] and depend on many different factors related to the patient, the specific characteristics of the causative agent [5,7,8], and errors produced during the management of those infections [9].

The most common pathogens in PJIs are *Staphylococcus aureus* and coagulase-negative staphylococci, followed by streptococci, enterococci, Gram-negative bacilli and anaerobes [1,2]. Anaerobic bacteria are involved in approximately 3% to 6% of PJIs [10], with *Cutibacterium acnes* (formerly known as *Propionibacterium acnes*) being the most frequently isolated species [11]. *C. acnes* is a Gram-positive anaerobic rod and a prevalent member of the common human skin microbiota. It was found to be frequent in some healthcare-related infections, especially in PJIs of the shoulder [12,13]. However, there is an ongoing debate if a *C. acnes*-positive culture obtained from a clinical specimen (always) indicates a true infection with this bacterium; there is a risk of skin-derived contamination during surgery and/or specimen handling and procession that could result in the cultivation of skin-resident *C. acnes* [14,15,16]. Moreover, in one study *C. acnes* was detected as a commensal of the native shoulder microbiome [17]. One possibility to distinguish between true infection, benign commensalism and contamination is to investigate the bacterial isolates by molecular methods, such as whole genome sequencing (WGS), in order to potentially demarcate PJI-associated types of *C. acnes* from types that are associated with disease-free sites.

Based on phylogenetic typing methods, the *C. acnes* population has been divided into six main phylotypes: IA_1_, IA_2_, IB, IC, II and III [18,19,20,21]. The different types are associated with different clinical conditions and normal skin [20,21,22,23,24,25,26,27]. Type IA_1_ is predominantly found in skin and it is, together with strains of phylotype IA_2_, also most commonly involved in moderate to severe acne, whereas types IB and II are often reported to be the predominant phylotypes associated with blood, soft tissue and medical device-related infections [20,21,22,23,24,25,26,27]. Type III, found frequently on the skin of the lower trunk, was reported to be associated with spinal disc infections. Based on the *C. acnes* core genome phylogeny, an SLST scheme was developed with a more detailed resolution, distinguishing ten main types, SLST types A, B, C, D, E, F, G, H, K, and L [22]. SLST types A–E correspond to phylotype IA_1_ strains, whereas SLST types F, G, H, K and L correspond to phylotypes IA_2_, IC, IB, II and III, respectively.

The aim of this study was to sequence, type and compare *C. acnes* strains isolated from healthy skin and PJIs that originated from seven different European hospitals. The PJI cases were divided into those with good treatment outcome and those with infection relapse. We show by WGS that there was no clear separation of skin isolates from PJI isolates. Interestingly, genomic differences beyond the phylotype level were detected in strains isolated in different hospitals from different countries.

## 2. Materials and Methods

### 2.1. Strain Isolation and Growth Conditions

Bacterial strains used in this study are listed in Table 1. Twelve strains were isolated from PJIs in seven European hospitals as part of a multicenter study supported by the European Study Group for Implant-Associated Infections (ESGIAI) of the European Society of Clinical Microbiology and Infectious Diseases (ESCMID), including six from patients with a good outcome and six from patients with relapse infection. The isolates were obtained from osteoarticular samples processed in the clinical microbiology laboratories according to internationally accepted methods [1]. *C. acnes* isolates from healthy skin were collected in Madrid (Spain), using a cotton swab from the alar and retroauricular creases. *C. acnes* isolates were grown on reinforced clostridial agar (Oxoid, Thermo Fisher Scientific, Waltham, USA) under anaerobic conditions at 37 °C for 3–4 days.

This study was approved by the ERC from Fundación Jiménez Díaz University Hospital, Madrid, Spain and Division of Infectious Diseases and Hospital Epidemiology, University Hospital of Zürich, Switzerland.

### 2.2. DNA Isolation and Genome Sequencing

Genomic DNA isolation of 16 *C. acnes* strains was performed using the MasterPure DNA purification kit (Epicentre). Concentration and purity of the isolated DNA was first checked with a Nanodrop ND-1000 (PeqLab Erlangen, Germany) and exact concentration was determined using the Qubit^®^ dsDNA HS Assay Kit, as recommended by the manufacturer (Life Technologies GmbH, Darmstadt, Germany). Illumina shotgun libraries were prepared using the Nextera XT DNA Sample Preparation Kit and subsequently sequenced on a MiSeq system with the reagent kit v3 with 600 cycles (Illumina, San Diego, CA, USA), as recommended by the manufacturer. Quality filtering was conducted with Trimmomatic version 0.36 [28]. On average, 1,964,279 paired-end reads (range: 1,474,132–2,400,076 reads) with an average read length of 2,526,079 bp (range: 2,459,078–2,604,163 bp) were used for the assemblies. The assembly was performed with the SPAdes genome assembler software version 3.13.0 [29]. The assembly was validated, and the sequence coverage determined with QualiMap version 2.2.1 [30]. The average coverage was 174-fold (range: 133–213-fold). All genome sequences are stored in GenBank. The accession numbers are listed in Table 1.

### 2.3. Bioinformatics Tools and Analyses

Gene prediction and the annotation of all genomes were performed with PGAP [31]. For phylogenomic analyses, the core genome was identified and aligned with Parsnp, a program which is part of the Harvest software package [32]. A total of 150 *C. acnes* genomes stored in GenBank were used, along with the 16 genomes sequenced here to build a core-genome-based phylogeny. The 150 genomes were selected according to their assembly quality (N_50_ > 500 kb). Reliable core-genome SNPs identified by Parsnp were used for the reconstruction of whole-genome phylogeny. Phylogenetic trees were visualized using the Interactive Tree of Life (iTOL; https://itol.embl.de/, accessed on 15 March 2021). For comparative genome analyses and visualization, the programs ACT [33] and BRIG were used [34]. SLST assignment was performed on http://medbac.dk/slst/pacnes, accessed on 1 March 2021. ResFinder [35] was used to identify (acquired) genes mediating antimicrobial resistance.

## 3. Results

### 3.1. Strain Cohort

Sixteen *C. acnes* strains were included in this study. These included twelve strains from PJIs (seven shoulder PJIs, four hip PJIs and one knee PJI) from seven different hospitals in five European countries, and four strains from healthy skin (from Spain) (Table 1). Among PJI-associated strains, six belonged to cases with a good outcome and six cases had an infection relapse (Supplementary Table S1). All cases, including relapse cases, were previously treated with surgical debridement and antibiotics [36]. Infection relapse was defined when persisting signs or symptoms of infection (pain, swelling, redness, wound secretion, elevated serum inflammatory parameters) were present after surgical debridement and antibiotic treatment, and two samples from periprosthetic tissue were positive for *C. acnes* based on conventional culture methods.

### 3.2. Typing and Whole Genome Phylogeny of C. acnes Strains

The genomes of the sixteen strains were sequenced; WGS results are summarized in Table 1. Regarding the assignment to the main six phylotypes, all four isolates from the skin belonged to the phylotype IA and could be further assigned to SLST types A1 (IA_1_), D1 (IA_1_), and F4 (IA_2_). In contrast, additional phylotypes were found in PJI cases, i.e., five type IB strains (all with the SLST type H1), four type IA_1_ strains (SLST types: two A1, one C1, one D1), two type II strains (SLST types K1 and a new K type), and one IA_2_ strain (SLST type F4). Thus, a total of seven SLST types were encountered among the PJI isolates, including six previously known SLST types and a new K type. The SLST type H1 was the most abundant type (66.7%) in PJI relapse cases.

**Figure 1:**
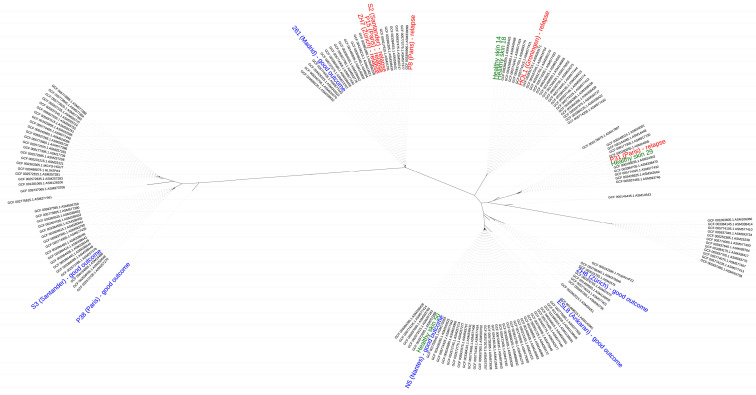
Phylogenomic comparison of *C. acnes* strains. The phylogeny was reconstructed from a core genome alignment and the comparison of high-quality SNPs. Strains isolated from healthy skin are labeled in green, strains from PJIs with a good outcome are in blue, and those from treatment failure (infection relapse) are in red. Labeled in black are genome-sequenced strains taken from GenBank (only genomes with high-quality assemblies (N_50_ > 500 kb); status March 2021). The geographical origin of the strains used in this study is indicated in brackets. The sixteen strains belong to different clades, indicating that there is not a clear separation according to their disease association, disease condition and geographical origin.

The genome sequences of the sixteen strains obtained in this study and 150 high-quality (N_50_ > 500 kb) genome sequences of *C. acnes* strains available at GenBank were phylogenetically analyzed by calling single nucleotide polymorphisms (SNPs) within the core genome using the tool Parsnp [32]. The phylogenomic analysis revealed that there was not an obvious separation of strains based on their disease association (healthy skin versus PJI), their disease condition (good outcome versus relapse) or on their geographical origin (Figure 1).

### 3.3. Genomic Differences beyond the Phylotype Level Due to the Presence of a Plasmid

**Figure 2:**
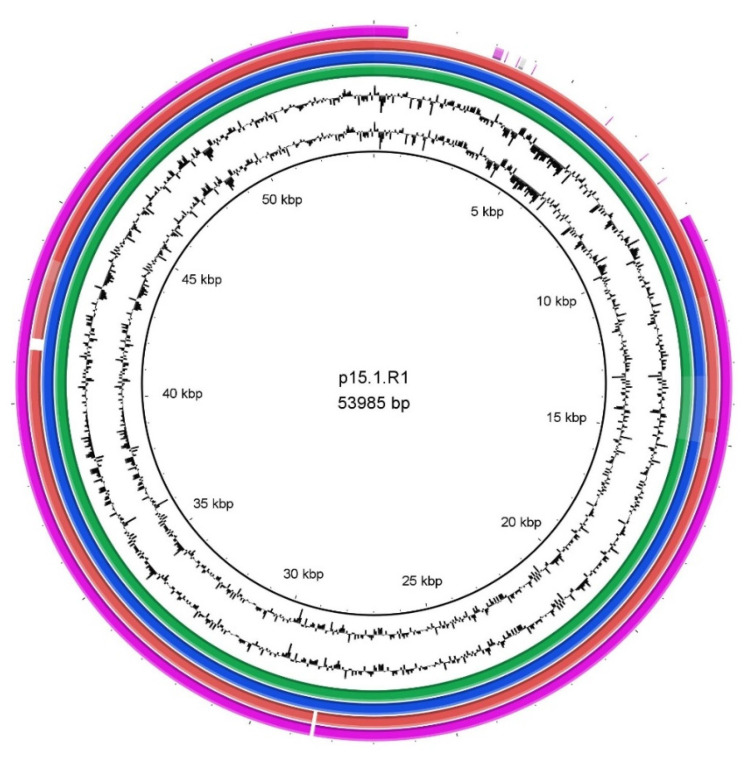
Comparison of a linear plasmid of *C. acnes*. The linear plasmid p15.1.R1 was used as a reference. The plasmid sequence was found in the strains ESL8 (green), HS29 (blue), 261 (red) and ZH8 (purple). Each strain belonged to a different SLST type (Table 1). A high synteny of the plasmid was detected in all four strains, which the exception of the plasmid in the ZH8 strain that had a deletion of approximately 10 kb.

Differences in genome sizes were noticed, and we thus decided to search all genomes for the presence of a linear plasmid, designated p15.1.R1 [37] or pIMPLE-HL096PA1 [38]. The plasmid was previously found in some *C. acnes* strains associated with acne vulgaris [38], and in several type II strains associated with prostate cancer [39]. The analysis revealed that the 54 kb plasmid was found in four strains, all of which belonged to different SLST types, including three isolates from PJIs with a good outcome (SLST types A1, C1, H1) and one isolate from healthy skin (SLST type F4). A comparative analysis of the plasmids using the tool BRIG (Figure 2) showed a deletion of approximately 10 kb in the plasmid of the C1 strain ZH8; this deletion was not detected before in any known plasmid sequence. Another plasmid, designated pTZC1, conferring resistance to macrolides, clindamycin, and tetracyclines, which has been found in *C. acnes* strains isolated in Japan, could not be identified in any of the 16 strains sequenced here [40].

### 3.4. Genomic Differences beyond the Phylotype Level Due to Other Genomic Islands

Further comparative investigations of genomes of strains belonging to the same SLST type were conducted to evaluate the basis of genome size differences, independent of the 54 kb linear plasmid. Three strains with additional genome content were identified: the F4 strain HS29 (from Spain), the H1 strain 261 (from Spain) and the A1 strain ESL8 (from Slovenia).

The F4 strain HS29 and the H1 strain 261 harbored transposase elements that were not found in the other F4 and H1 strains, respectively. The A1 strain ESL8 isolated in Slovenia comprised some elements that are not present in the other sequenced A1 strains from Nantes and Madrid, such as an arsenic resistance protein, negative regulator of beta-lactam expression, zinc ribbon domain protein, and genes involved in siderophore biosynthesis and cadmium resistance.

No acquired genes mediating antimicrobial resistance were identified in *C. acnes* strains associated with PJIs. Only strain HS29, isolated from healthy skin, harbored the resistance gene *erm*(X), which confers resistance to macrolides and lincosamides [41].

## 4. Discussion

This study aimed for the genomic analysis of a cohort of *C. acnes* strains that are associated with PJI cases and healthy skin. The study serves as a proof-of-concept and basis for a large-cohort study that includes different hospitals across Europe. We sequenced two different groups of PJI-associated strains, selected on the basis of the clinical outcome, i.e., a good outcome or treatment failure (infection relapse). The study should also clarify if the *C. acnes* genomes sequenced here contain any gene content that might be relevant for PJIs, and that has not been identified before.

Over the years, typing systems for *C. acnes* have gained more importance to establish an association between the different phylotypes and clinical conditions. Several studies have shown that the most frequent phylotypes associated with PJIs are phylotypes IB and II [20,42]. However, other studies have also reported the involvement of strains of the phylotype IA1 in PJIs [43,44]. In our PJI strain cohort, the most predominant phylotypes were IB and IA1, more specifically the SLST type H1 (41.6%) and the SLST types A1, C1, and D1 (33.3% in total). In the hip PJI cases, three out of four isolates belonged to H1, whereas shoulder PJI cases exhibited a more diverse set of strains (A1, D1, F4, two H1, K1, K_new_).

Notable is the dominance of the SLST type H1 in relapse cases (4/6), in particular in hip PJIs, although more studies with larger cohorts are needed to confirm this. In a previous study, H1 strains were detected in deep tissue specimens retrieved during revision shoulder arthroplasty in monoclonal cultures [45,46]. In contrast, in the study of Liew-Littorin et al., the most frequent SLST type among PJI isolates was A1 followed by D1; in their study, A1 was the most abundant SLST type in shoulder, hip, and knee infections [43]. In our cohort, H1 was the predominant SLST type in hip infections, whereas in shoulder infections no predominance of a specific type could be detected. These observed differences could indicate that phylogenetically distinct strains of *C. acnes* have the potential to cause PJIs. Alternatively, infections may not always be monoclonal (i.e., caused by one *C. acnes* clone). A recent study found that multiple phylotypes of *C. acnes* within deep tissue specimens were retrieved during revision shoulder arthroplasty in about 50% of cases, indicative of polyclonal/mixed infections, i.e., infections caused by multiple strains belonging to different *C. acnes* phylotypes [46]. Furthermore, in some cases there might be one major infectious *C. acnes* clone and a minor contaminant present; the latter could be introduced during surgery or sample acquisition/procession. One study showed that *C. acnes* can be isolated unexpectedly from intraoperative samples from patients with mild symptoms, and even from asymptomatic patients [14]. Further studies that investigate the clonality of strains from specimens with suspected PJIs and the skin of these patients are needed to evaluate the disease etiology of specific *C. acnes* clones/phylotypes. The issue is further complicated in light of growth differences between different *C. acnes* phylotypes; in particular, type IB strains usually grow more slowly than type IA strains on common agar media (Brüggemann, personal communication). To address these issues, there is a need to isolate and type multiple strains from primary cultures, but this is currently not routinely performed. Alternatively, a culture-independent SLST amplicon sequencing approach could be applied which is able to determine the relative abundancies of all *C. acnes* phylotypes in a given sample [22].

Regarding strains isolated from healthy skin, the four strains analyzed here all belonged to phylotype IA, confirming that this phylotype is the most abundant on human skin [20,25,26,47].

We analyzed the genome content of the 16 strains sequenced here and compared them to previously sequenced genomes. The genomes were highly similar to previously sequenced genomes of *C. acnes* (Figure 1). Most of them belonged to phylotype I; strains S3 and P38 were the only ones that belonged to phylotype II.

A linear plasmid, previously described by Brüggemann et al. and Kasimatis et al. [37,38], was found in four isolates. This plasmid of approximately 54 kb contains a gene locus for tight adherence (tad) that codes for the biosynthesis of adhesive Flp (fimbrial low-molecular weight protein) pili, that are predicted to be involved in enhancing attachment and, potentially, biofilm formation [38,39]. The plasmid-positive strains belonged to four different SLST types, which shows that its presence is not phylotype-dependent. Interestingly, the presence of the plasmid did not correlate with disease association; the plasmid was found in one strain isolated from healthy skin, and three strains from PJI cases with a good outcome. We did not identify any plasmid in those strains that were isolated from infection relapse cases. In the study of Liew-Littorin et al., the plasmid was found at the same frequency in samples from PJIs and samples from the skin [43], underlining that the plasmid is not a specific marker for PJI-associated strains.

Besides the plasmid, three strains (HS29, 261 and ESL8) belonging to different SLST types (F4, H1 and A1, respectively) with additional genome content were identified. Interestingly, the geographic origin of these strains differed from other sequenced strains assigned to the same SLST types, i.e., the F4 strain P31 (from Paris), four H1 strains (from Santander, Paris and Zürich) and two A1 strains (from Nantes and Madrid). Thus, strains belonging to the same SLST type but isolated from different geographic locations can differ in their flexible genome, indicative of microevolution, possibly by horizontal gene transfer.

Overall, our results revealed that *C. acnes* isolates from patients with treatment success and failure were genetically similar, suggesting that treatment failure might be primarily related to the choice of antibiotics and clinical management, rather than dependent on the actual causative strain [36]. Similar results were seen in a study with *S. aureus* strains [48], in which isolates from patients with treatment success and failure were genetically very similar, indicating that treatment failure was associated with the presence of an antimicrobial-resistant phenotype and the use of non-biofilm-active antibiotic treatment.

This study has several limitations such as the small number of isolates included. In particular, the four-strain healthy cohort provides just a glimpse of the diversity of *C. acnes* types on normal human skin that is usually colonized by a myriad of strains belonging to multiple phylotypes. Besides adding more strains in a future study, a very interesting, unanswered question is also whether the strains isolated from the primary infection and the relapse infection are identical or represent different strains/phylotypes. The former could indicate a clear relapse with the same strain (due to surgery or treatment failure); the latter could indicate a secondary infection or a superinfection. However, external factors (such as surgical treatment, characteristics of the patients, and others) are also involved in the evolution, and probably it is the sum of those with intrinsic characteristics of the isolates that decides the outcome. In addition, another limitation might be the involvement of different hospitals in different countries; this increases the risk of a bias because of different processes of clinical and/or data management.

## Figures and Tables

**Table 1:** Information regarding whole genome sequencing data of sixteen *C. acnes* strains.

Strain	GenBank	Source	Joint Site	Phylotype	SLST	Size (bp)	Coverage	Contigs	N50 (bp)	Plasmid	Geographical Origin
ZH7	JAGDNT000000000	Relapse PJI	Hip	IB	H1	2,544,272	203	17	323,469	-	Zürich, Switzerland
P8	JAGDNS000000000	Relapse PJI	Shoulder	IB	H1	2,545,631	169	19	168,849	-	Paris, France
P15	JAGDNR000000000	Relapse PJI	Hip	IB	H1	2,544,110	193	16	572,713	-	Paris, France
P31	JAGDNQ000000000	Relapse PJI	Shoulder	IA	F4	2,482,181	171	14	690,112	-	Paris, France
S2	JAGDNP000000000	Relapse PJI	Hip	IB	H1	2,545,388	183	19	337,942	-	Santander, Spain
HOL1	JAGDNO000000000	Relapse PJI	Shoulder	IA	D1	2,535,867	178	16	738,597	-	Groningen, Netherlands
261	JAGDNN000000000	PJI	Shoulder	IB	H1	2,604,163	133	25	323,553	54.2	Madrid, Spain
ZH8	JAGDNM000000000	PJI	Hip	IA	C1	2,526,283	187	20	302,006	46.1	Zürich, Switzerland
S3	JAGDNL000000000	PJI	Shoulder	II	K_new_	2,478,438	207	9	725,650	-	Santander, Spain
P38	JAGDNK000000000	PJI	Shoulder	II	K1	2,459,078	135	12	523,312	-	Paris, France
ESL8	JAGDNJ000000000	PJI	Knee	IA	A1	2,579,590	133	22	400,190	54.0	Ankaran, Slovenia
N5	JAGDNI000000000	PJI	Shoulder	IA	A1	2,478,335	168	14	344,049	-	Nantes, France
HS14	JAGDNH000000000	Skin	-	IA	D1	2,536,118	187	10	738,783	-	Madrid, Spain
HS18	JAGDNG000000000	Skin	-	IA	D1	2,535,241	213	14	700,603	-	Madrid, Spain
HS29	JAGDNF000000000	Skin	-	IA	F4	2,543,557	160	24	280,277	52.6	Madrid, Spain
HS50	JAGDNE000000000	Skin	-	IA	A1	2,479,007	163	10	738,366	-	Madrid, Spain

## Data Availability

All 16 genome sequences obtained in this study are stored in GenBank. The accession numbers are listed in Table 1.

## Supplementary Materials

The following are available online at https://www.mdpi.com/article/10.3390/microorganisms9071500/s1, Table S1: Clinical data regarding *C. acnes* isolates.

## References

[1] Tande A.J., Patel R. (2014). Prosthetic Joint Infection. Clin. Microbiol. Rev..

[2] Zimmerli W., Trampuz A., Ochsner P.E. (2004). Prosthetic-Joint Infections. N. Engl. J. Med..

[3] Osmon D.R., Berbari E.F., Berendt A.R., Lew D., Zimmerli W., Steckelberg J.M., Rao N., Hanssen A., Wilson W.R. (2013). Diagnosis and Management of Prosthetic Joint Infection: Clinical Practice Guidelines by the Infectious Diseases Society of America. Clin. Infect. Dis..

[4] Li C., Renz N., Trampuz A. (2018). Management of Periprosthetic Joint Infection. Hip Pelvis.

[5] Kandel C.E., Jenkinson R., Daneman N., Backstein D., Hansen B.E., Muller M.P., Katz K.C., Widdifield J., Bogoch E., Ward S. (2019). Predictors of Treatment Failure for Hip and Knee Prosthetic Joint Infections in the Setting of 1- and 2-Stage Exchange Arthroplasty: A Multicenter Retrospective Cohort. Open Forum Infect. Dis..

[6] Yaghmour K.M., Chisari E., Khan W.S. (2019). Single-Stage Revision Surgery in Infected Total Knee Arthroplasty: A PRISMA Systematic Review. J. Clin. Med..

[7] Jhan S.-W., Lu Y.-D., Lee M.S., Lee C.-H., Wang J.-W., Kuo F.-C. (2017). The Risk Factors of Failed Reimplantation Arthroplasty for Periprosthetic Hip Infection. BMC Musculoskelet. Disord..

[8] Masters J.P., Smith N.A., Foguet P., Reed M., Parsons H., Sprowson A.P. (2013). A Systematic Review of the Evidence for Single Stage and Two Stage Revision of Infected Knee Replacement. BMC Musculoskelet. Disord..

[9] Li C., Renz N., Trampuz A., Ojeda-Thies C. (2020). Twenty Common Errors in the Diagnosis and Treatment of Periprosthetic Joint Infection. Int. Orthop..

[10] Shah N.B., Tande A.J., Patel R., Berbari E.F. (2015). Anaerobic Prosthetic Joint Infection. Anaerobe.

[11] Benito N., Franco M., Ribera A., Soriano A., Rodriguez-Pardo D., Sorlí L., Fresco G., Fernández-Sampedro M., Dolores del Toro M., Guío L. (2016). Time Trends in the Aetiology of Prosthetic Joint Infections: A Multicentre Cohort Study. Clin. Microbiol. Infect..

[12] Achermann Y., Goldstein E.J.C., Coenye T., Shirtliffa M.E. (2014). Propionibacterium Acnes: From Commensal to Opportunistic Biofilm-Associated Implant Pathogen. Clin. Microbiol. Rev..

[13] Portillo M.E., Corvec S., Borens O., Trampuz A. (2013). Propionibacterium Acnes: An Underestimated Pathogen in Implant-Associated Infections. BioMed Res. Int..

[14] Dorrestijn O., Pruijn N. (2021). Reply: Low-Grade *Cutibacterium Acnes* Shoulder Infections Do Exist!: In Response to the Letter to the Editor by Reinier WA Spek, Job N Doornberg, David Ring and Michel PJ van Den Bekerom. Shoulder Elb..

[15] Pruijn N., Schuncken A.C., Kosse N.M., Hofstad C.J., Dorrestijn O. (2021). Pre- and Peroperative Diagnosis of *Cutibacterium Acnes* Infections in Shoulder Surgery: A Systematic Review. Shoulder Elb..

[16] Spek R.W., Doornberg J.N., Ring D., van den Bekerom M.P. (2021). Can Surgeons Differentiate between Painful Shoulders That Grow *Cutibacterium Acnes* and Infection Benefitting from Treatment?. Shoulder Elb..

[17] Hudek R., Brobeil A., Brüggemann H., Sommer F., Gattenlöhner S., Gohlke F. (2021). Cutibacterium Acnes Is an Intracellular and Intra-Articular Commensal of the Human Shoulder Joint. J. Shoulder Elb. Surg..

[18] McDowell A., Valanne S., Ramage G., Tunney M.M., Glenn J.V., McLorinan G.C., Bhatia A., Maisonneuve J.-F., Lodes M., Persing D.H. (2005). Propionibacterium Acnes Types I and II Represent Phylogenetically Distinct Groups. J. Clin. Microbiol..

[19] McDowell A., Perry A.L., Lambert P.A., Patrick S. (2008). A New Phylogenetic Group of Propionibacterium Acnes. J. Med. Microbiol..

[20] McDowell A., Barnard E., Nagy I., Gao A., Tomida S., Li H., Eady A., Cove J., Nord C.E., Patrick S. (2012). An Expanded Multilocus Sequence Typing Scheme for Propionibacterium Acnes: Investigation of ‘Pathogenic’, ‘Commensal’ and Antibiotic Resistant Strains. PLoS ONE.

[21] Kilian M., Scholz C.F.P., Lomholt H.B. (2012). Multilocus Sequence Typing and Phylogenetic Analysis of Propionibacterium Acnes. J. Clin. Microbiol..

[22] Scholz C.F.P., Jensen A., Lomholt H.B. (2014). A Novel High-Resolution Single Locus Sequence Typing Scheme for Mixed Populations of Propionibacterium Acnes In Vivo. PLoS ONE.

[23] Yu Y., Champer J., Garbán H., Kim J. (2015). Typing of *Propionibacterium Acnes*: A Review of Methods and Comparative Analysis. Br. J. Dermatol..

[24] McDowell A., Nagy I., Magyari M., Barnard E., Patrick S. (2013). The Opportunistic Pathogen Propionibacterium Acnes: Insights into Typing, Human Disease, Clonal Diversification and CAMP Factor Evolution. PLoS ONE.

[25] Petersen R.L.W., Scholz C.F.P., Jensen A., Brüggemann H., Lomholt H.B. (2017). *Propionibacterium Acnes* Phylogenetic Type III Is Associated with Progressive Macular Hypomelanosis. Eur. J. Microbiol. Immunol..

[26] Lomholt H.B., Kilian M. (2010). Population Genetic Analysis of Propionibacterium Acnes Identifies a Subpopulation and Epidemic Clones Associated with Acne. PLoS ONE.

[27] Brüggemann H., Salar-Vidal L., Gollnick H.P.M., Lood R. (2021). A Janus-Faced Bacterium: Host-Beneficial and -Detrimental Roles of Cutibacterium Acnes. Front. Microbiol..

[28] Bolger A.M., Lohse M., Usadel B. (2014). Trimmomatic: A Flexible Trimmer for Illumina Sequence Data. Bioinformatics.

[29] Bankevich A., Nurk S., Antipov D., Gurevich A.A., Dvorkin M., Kulikov A.S., Lesin V.M., Nikolenko S.I., Pham S., Prjibelski A.D. (2012). SPAdes: A New Genome Assembly Algorithm and Its Applications to Single-Cell Sequencing. J. Comput. Biol..

[30] García-Alcalde F., Okonechnikov K., Carbonell J., Cruz L.M., Götz S., Tarazona S., Dopazo J., Meyer T.F., Conesa A. (2012). Qualimap: Evaluating next-Generation Sequencing Alignment Data. Bioinformatics.

[31] Tatusova T., DiCuccio M., Badretdin A., Chetvernin V., Nawrocki E.P., Zaslavsky L., Lomsadze A., Pruitt K.D., Borodovsky M., Ostell J. (2016). NCBI Prokaryotic Genome Annotation Pipeline. Nucleic Acids Res..

[32] Treangen T.J., Ondov B.D., Koren S., Phillippy A.M. (2014). The Harvest Suite for Rapid Core-Genome Alignment and Visualization of Thousands of Intraspecific Microbial Genomes. Genome Biol..

[33] Carver T.J., Rutherford K.M., Berriman M., Rajandream M.-A., Barrell B.G., Parkhill J. (2005). ACT: The Artemis Comparison Tool. Bioinformatics.

[34] Alikhan N.-F., Petty N.K., Ben Zakour N.L., Beatson S.A. (2011). BLAST Ring Image Generator (BRIG): Simple Prokaryote Genome Comparisons. BMC Genomics.

[35] Bortolaia V., Kaas R.S., Ruppe E., Roberts M.C., Schwarz S., Cattoir V., Philippon A., Allesoe R.L., Rebelo A.R., Florensa A.F. (2020). ResFinder 4.0 for Predictions of Phenotypes from Genotypes. J. Antimicrob. Chemother..

[36] Kusejko K., Auñón Á., Jost B., Natividad B., Strahm C., Thurnheer C., Pablo-Marcos D., Slama D., Scanferla G., Uckay I. (2020). The Impact of Surgical Strategy and Rifampin on Treatment Outcome in *Cutibacterium* Periprosthetic Joint Infections. Clin. Infect. Dis..

[37] Brüggemann H., Lomholt H.B., Tettelin H., Kilian M. (2012). CRISPR/Cas Loci of Type II Propionibacterium Acnes Confer Immunity against Acquisition of Mobile Elements Present in Type I P. Acnes. PLoS ONE.

[38] Kasimatis G., Fitz-Gibbon S., Tomida S., Wong M., Li H. (2013). Analysis of Complete Genomes of *Propionibacterium Acnes* Reveals a Novel Plasmid and Increased Pseudogenes in an Acne Associated Strain. BioMed Res. Int..

[39] Davidsson S., Carlsson J., Mölling P., Gashi N., Andrén O., Andersson S.-O., Brzuszkiewicz E., Poehlein A., Al-Zeer M.A., Brinkmann V. (2017). Prevalence of Flp Pili-Encoding Plasmids in Cutibacterium Acnes Isolates Obtained from Prostatic Tissue. Front. Microbiol..

[40] Aoki S., Nakase K., Nakaminami H., Wajima T., Hayashi N., Noguchi N. (2019). Transferable Multidrug-Resistance Plasmid Carrying a Novel Macrolide-Clindamycin Resistance Gene, *Erm* (50), in *Cutibacterium Acnes*. Antimicrob. Agents Chemother..

[41] Ross J.I., Eady E.A., Carnegie E., Cove J.H. (2002). Detection of Transposon Tn5432–Mediated Macrolide-Lincosamide-Streptogramin B (MLSB) Resistance in Cutaneous Propionibacteria from Six European Cities. J. Antimicrob. Chemother..

[42] Aubin G.G., Lavigne J.-P., Foucher Y., Dellière S., Lepelletier D., Gouin F., Corvec S. (2017). Tropism and Virulence of Cutibacterium (Formerly Propionibacterium ) Acnes Involved in Implant-Associated Infection. Anaerobe.

[43] Liew-Littorin C., Brüggemann H., Davidsson S., Nilsdotter-Augustinsson Å., Hellmark B., Söderquist B. (2019). Clonal Diversity of Cutibacterium Acnes (Formerly Propionibacterium Acnes) in Prosthetic Joint Infections. Anaerobe.

[44] El Sayed F., Roux A.-L., Sapriel G., Salomon E., Bauer T., Gaillard J.-L., Rottman M. (2019). Molecular Typing of Multiple Isolates Is Essential to Diagnose Cutibacterium Acnes Orthopedic Device–Related Infection. Clin. Infect. Dis..

[45] Sampedro M.F., Piper K.E., McDowell A., Patrick S., Mandrekar J.N., Rouse M.S., Steckelberg J.M., Patel R. (2009). Species of Propionibacterium and Propionibacterium Acnes Phylotypes Associated with Orthopedic Implants. Diagn. Microbiol. Infect. Dis..

[46] Bumgarner R.E., Harrison D., Hsu J.E. (2019). *Cutibacterium Acnes* Isolates from Deep Tissue Specimens Retrieved during Revision Shoulder Arthroplasty: Similar Colony Morphology Does Not Indicate Clonality. J. Clin. Microbiol..

[47] Dréno B., Dagnelie M.A., Khammari A., Corvec S. (2020). The Skin Microbiome: A New Actor in Inflammatory Acne. Am. J. Clin. Dermatol..

[48] Wildeman P., Tevell S., Eriksson C., Lagos A.C., Söderquist B., Stenmark B. (2020). Genomic Characterization and Outcome of Prosthetic Joint Infections Caused by Staphylococcus Aureus. Sci. Rep..

