# Supplementary material for: Genomic Analysis of Cutibacterium acnes Strains Isolated from Prosthetic Joint Infections
Source: Microorganisms. 2021 Jul 14;9(7):1500. doi: 10.3390/microorganisms9071500 (PMC8307888; doi:10.3390/microorganisms9071500)
Supplement: Supplementary file 1 [file microorganisms-09-01500-s001.zip › microorganisms-1277137-supplementary.pdf]

Table S1. Clinical data regarding PJI *C. acnes* isolates included in this study.

Strain	Source	Joint site	Age*	Sex	Immuno-suppressed
ZH7	Relapse PJI	Hip	64	Male	No
P8	Relapse PJI	Shoulder	46	Male	Solid tumor
P15	Relapse PJI	Hip	79	Male	No
P31	Relapse PJI	Shoulder	37	Male	No
S2	Relapse PJI	Hip	76	Female	No
HOL1	Relapse PJI	Shoulder	76	Male	No
261	PJI	Shoulder	53	Male	No
ZH8	PJI	Hip	62	Female	No
S3	PJI	Shoulder	62	Male	No
P38	PJI	Shoulder	70	Male	No
ESL8	PJI	Knee	59	Male	No
N5	PJI	Shoulder	34	Male	No

*Age of the patient at the time of infection.
